# The Gray Zone of H-Reflex in Runners: When Should We Suspect Pathology? A Pilot Study

**DOI:** 10.3390/jcm15031297

**Published:** 2026-02-06

**Authors:** L. H. M. P. De Silva, Andriy Maznychenko, Andriy Gorkovenko, Olena Kolosova, Tetiana Abramovych, Oleh V. Vlasenko, Vasyl Melenko, Oleksii Sulyma, Tetyana Poruchynska, Inna Sokolowska

**Affiliations:** 1 Department of Physical Education, Gdansk University of Physical Education and Sport, Kazimierza Gorskiego Str. 1, 80-336 Gdansk, Poland; 2 Department of Sports Science, University of Sri Jayewardenepura, Nugegoda 10250, Sri Lanka; 3 Department of Movement Physiology, Bogomoletz Institute of Physiology NAS of Ukraine, Bogomoletz Str. 4, 01024 Kyiv, Ukraine; 4 Scientific Research Center, National University of Ukraine on Physical Education and Sport, Fizkultury Str. 1, 03150 Kyiv, Ukraine; 5 Department of Biomedical Foundations of Physical Education and Physical Rehabilitation, Vinnytsia Mykhailo Kotsiubynskyi State Pedagogical University, Ostroz’koho Str. 32, 21001 Vinnytsia, Ukraine; 6 Department of Spinal Surgery, State Institution “Institute of Traumatology and Orthopedics”, National Academy of Medical Science of Ukraine, Bulvarno-Kudriavska Str. 27, 01054 Kyiv, Ukraine; 7 Department of Anatomy, Normal and Pathological Physiology, Lesia Ukrainka Volyn National University, Voli Ave. 13, 43025 Lutsk, Ukraine

**Keywords:** spinal excitability, H-reflex modulation, fatigue, neuromuscular adaptation, sports diagnostics

## Abstract

**Background/Objectives**: Spinal excitability may undergo adaptive modulation in response to training load, sport-specific demands, and fatigue. While high-impact sports are known to influence reflex responsiveness, the extent to which these changes differ from athletes in lower-impact disciplines remains unclear. This study aimed to investigate post-exercise changes in Hmax/Mmax ratio among trained runners with varied sport backgrounds, and to identify emergent physiological profiles that may reflect differential spinal adaptation to fatigue. **Methods**: Twenty-two trained athletes underwent unilateral H-reflex testing before and after treadmill running performed to voluntary exhaustion. Amplitudes of the H-reflex and M-wave were recorded, and Hmax/Mmax ratios were analyzed. Based on a physiologically relevant threshold commonly used to distinguish normal from suppressed reflex amplitudes, participants were post hoc classified into three groups: Group A (pre- and post-test ratios above threshold), Group B (pre above, post below), and Group C (both below). A two-way repeated-measures ANOVA was used to assess between-group effects. **Results**: Significant differences were found across groups and conditions (*p* < 0.001). Group A maintained reflex ratios above the threshold, indicating stable excitability. Group B showed the greatest suppression (approximately 66%), transitioning from normal to subthreshold values. Group C remained consistently below-threshold. A significant interaction (*p* < 0.0001) confirmed that reflex modulation varied by physiological profile. A small but statistically significant reduction in H-reflex latency was also observed; however, this change remained within normal physiological variability. **Conclusions**: Postexercise H-reflex modulation revealed heterogeneous neuromuscular responses among athletes. These findings may contribute to understanding how sport-specific demands and fatigue shape spinal excitability and may help identify individuals with adaptive or potentially pathological profiles relevant to sports diagnostics.

## 1. Introduction

The Hoffmann reflex (H-reflex) is one of the few tools that allows direct access to spinal excitability in an intact human. A brief electrical stimulus delivered to a peripheral nerve triggers a monosynaptic response whose amplitude reflects how the spinal cord filters and processes sensory input. When expressed as the Hmax/Mmax ratio, this response becomes a quantitative signature of the underlying afferent–motoneuron interaction and its sensitivity to presynaptic regulation [1,2,3]. Its non-invasive nature and responsiveness to physiological change have made the H-reflex a key method for investigating neuromuscular function not only in research [2,3,4] and clinical practice [5,6], but increasingly in sports science [1,7,8].

Athletes exposed to high training loads and repetitive mechanical stress demonstrate well-established neuromuscular adaptations, including changes in reflex sensitivity, motor unit recruitment strategies, and spinal plasticity [4,7,8]. These adaptations may optimize performance but may also mask early deviations from typical reflex behavior that arise from cumulative mechanical loading [9,10,11]. Variability in H-reflex amplitude and Hmax/Mmax ratios is therefore of interest not only from a performance perspective but also for identifying individuals whose neuromuscular profiles deviate from expected physiological ranges.

Previous clinical studies show that altered H-reflex parameters can reflect atypical neuromuscular processing under conditions such as polyneuropathy or unilateral radiculopathy, with abnormalities confirmed through imaging and standard diagnostics [5,6]. These findings do not imply pathology in athletes, but they do demonstrate that unusually low H-reflex responses can serve as indicators of atypical spinal excitability warranting closer examination.

Acute fatigue is known to suppress H-reflex amplitude, primarily through increased presynaptic inhibition and changes in afferent feedback from metabolically sensitive group III and IV fibers [12,13]. However, the magnitude of suppression varies considerably between individuals and may depend on training history and sport-specific mechanical loading. Understanding these individual response patterns may help identify athletes whose reflex excitability consistently falls near the lower end of the physiological spectrum.

Despite extensive use of the H-reflex in neurophysiology, relatively little is known about baseline Hmax/Mmax characteristics in trained runners and how these characteristics modulate the reflex response to exhaustive exercise. In particular, it is unclear whether athletes exhibit distinct reflex profiles that remain stable across fatigue conditions and whether individuals with unusually low ratios represent a functional variant or a potential early indicator of atypical spinal excitability.

Based on prior observations by Jankus et al. [13], values below approximately 0.4 fall near the lower bound of expected physiological variation and therefore provide a useful reference point for distinguishing different excitability profiles. In this study, athletes were classified into groups according to whether their Hmax/Mmax ratios remained above this boundary, crossed it after exercise, or remained consistently low.

We hypothesize that athletes participating in high-impact running events will exhibit alterations in the H-reflex parameters, which may reflect changes in spinal cord excitability associated with repetitive mechanical loading.

The aim of this study was to examine post-exercise changes in the Hmax/Mmax ratio in trained runners and to identify distinct patterns of reflex excitability, including individuals whose values fall within a functional “gray zone” near the lower limit of expected physiological variation.

## 2. Materials and Methods

### 2.1. Participants and Study Design

This pilot study involved 22 healthy trained runners (both male and female), aged between 18 and 23 years. Initially, 30 athletes were recruited; however, 8 participants were excluded due to the inability to obtain a technically reliable H-reflex signal. Reliability was assessed during the pre-exercise measurements and required: a clearly identifiable H-reflex and M-wave, stable waveform morphology across repeated stimuli, and predictable recruitment with increasing stimulus intensity. All participants were highly trained athletes, engaging in approximately six training sessions per week. Each had competitive experience at regional or national levels, and were members of the national team in their respective disciplines. The participants were free from diagnosed musculoskeletal disorders, neurological conditions, or recent injuries that could affect lower limb function.

All volunteers provided written informed consent after being informed of the study’s purpose and procedures. The study was approved by the Local Ethics Committee of the Gdansk University of Physical Education and Sport (NKBBN/628/2019) and conducted in accordance with the Declaration of Helsinki and its subsequent amendments. The study adhered to STROBE guidelines.

A priori power analysis performed with G*Power 3.1 software (α = 0.05, power = 0.80, effect size f = 0.88) indicated that a minimum of 18 participants would be required to detect a significant interaction effect. The final sample of 22 athletes exceeded this threshold, providing sufficient statistical power for the analysis.

Anthropometric data were recorded prior to testing using the InBody 720 body composition analyzer (InBody Co., Ltd., Seoul, Republic of Korea) and the Seca 213 portable stadiometer (seca GmbH & Co. KG, Hamburg, Germany). The average values were: BMI = 20.2 ± 1.9, height = 173.4 ± 8.8 cm, and weight = 62.2 ± 8.1 kg. Participants were instructed to avoid strenuous activity for 24 h before the experiment. Some individuals had transitioned into running from other sports, including high-impact activities such as basketball, gymnastics or soccer (see Table 1). The transition history of these participants was defined as the documented sequence of athletes’ prior sporting specializations before their current focus, including any change in sport type, event distance, or training modality.

All experimental sessions were conducted in a laboratory maintained at 20–22 °C, and this temperature remained stable throughout data collection. In addition, participants wore standard athletic clothing (T-shirts, shorts, and running shoes), which minimized the risk of either cooling or overheating during electrophysiological assessments. These controlled ambient conditions helped to reduce the potential influence of temperature on H-reflex amplitude.

The experiment was designed to assess spinal motoneuron excitability before and after exhaustive exercise. Each participant performed a treadmill run to volitional exhaustion using a motorized treadmill (h/p/cosmos Saturn, Germany). Volitional exhaustion was defined as the point at which the athlete could no longer maintain the required pace despite verbal encouragement.

The treadmill protocol adopted from Tomczyk et al. (2022) [14] involved progressive loading: after a 5 min warm-up walk at 5 km/h with a 1.5% incline, the running test progressed in 3 min stages, starting at 8 km/h and increasing to 12 km/h. Once 12 km/h was reached, the incline was increased sequentially to 5%, 10%, and 15% until exhaustion. Participants typically ran for approximately 25 min before reaching voluntary exhaustion. All athletes reached volitional exhaustion at a mean rating of perceived exertion (RPE) of 19.3 ± 0.6 on the Borg 6–20 scale.

**Figure 1:**
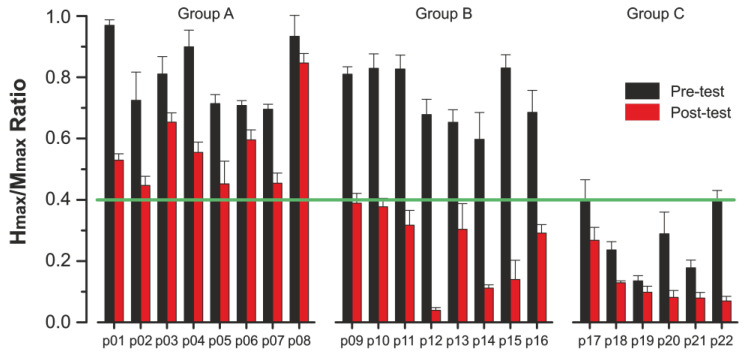
Individual Hmax/Mmax ratios for each participant before (black columns) and after (red columns) treadmill running. Green line indicates the threshold value of 0.40 used for group classification. Participants are grouped by reflex profile: Group A (stable) p01–p08, Group B (suppressed posttest) p09–p16, and Group C (consistently low) p17–p22. Values are presented as mean ± SEM.

Group divisions were determined post hoc, based on the Hmax/Mmax ratios: participants were divided into three groups using a threshold of 0.4, which reflects the lower bound of normal Hmax/Mmax ratios as established in prior normative studies [13]. Group A consisted of 8 athletes whose pre- and post-test Hmax/Mmax ratios remained above 0.4, indicating stable reflex excitability. Group B included 8 athletes who began with ratios above 0.4 but dropped below this threshold after exhaustive running, suggesting a transition into suppressed excitability. Group C comprised 6 athletes whose ratios were consistently below 0.4 both before and after exercise, reflecting persistently low spinal reflex responsiveness (Figure 1).

### 2.2. Electrophysiological Recording

All experimental sessions were conducted at the same time of day, between 10:00 and 12:00, to minimize the influence of circadian fluctuations on neuromuscular excitability. During the H-reflex registration, participants lay comfortably in the prone position with their eyes closed to ensure relaxation and to reduce variability associated with visual or postural inputs. The same body position and testing conditions were maintained for both pre-exercise and post-exercise measurements.

Electrophysiological recordings were taken at two time points: (1) baseline (pre-run) and (2) 15 min post-exercise. The primary outcome measure was the Hmax/Mmax ratio, derived from recordings of the H-reflex and M-response in the soleus muscle. These responses were elicited via bipolar electrical stimulation of the tibial nerve in the popliteal fossa. Surface EMG electrodes (Biopac EL503; BIOPAC Systems, Inc., Goleta, CA, USA) were placed along the mid-dorsal line of the leg, approximately 4 cm distal to the junction of the gastrocnemius heads with the Achilles tendon. Background muscle activity was continuously monitored using real-time EMG visualization in Spike2 v.9 (CED, Cannock, UK) software. Skin preparation included alcohol cleansing and abrasion. Electrode placement was confirmed via palpation during voluntary contractions, with a fixed interelectrode distance of 20 mm, following SENIAM guidelines.

Electrical stimulation was provided using rectangular-shaped pulses (a set of 10 stimuli, impulse duration 1 ms [1], 20 s between-impulse intervals). Stimulation was applied via an isolated constant-current stimulator (Digitimer DS3; Digitimer Limited, Welwyn Garden City, UK). To evoke both the H-reflex and M-wave, stimulus intensity was gradually increased. The maximal M-wave was determined at the beginning of the experiment and re-evoked at the end of the protocol to ensure consistency. Only data from participants with stable Mmax amplitudes (within ±5%) were included in the final analysis [15,16]. Signals were amplified (Brownlee model 440, Santa Clara, CA, USA), digitized at 10 kHz, and recorded with Spike2 v.9 software (CED, Cannock, UK).

### 2.3. Data Analysis

Electrophysiological data were processed using OriginPro v.10.1 (OriginLab Corporation, Northampton, MA, USA). Peak-to-peak amplitudes of the H-reflex and M-response were measured, and the Hmax/Mmax ratio was calculated for each participant at both time points. Mean values and standard error of mean (SEM) were computed to assess central tendency and variability. For the H-reflex, ten stimuli were delivered with 20 s interstimulus intervals to avoid post-activation depression. Trials contaminated by artifacts were discarded, and a minimum of 8 clean responses were retained and averaged for analysis. For the M-wave, three supramaximal stimuli were applied (also with 20 s intervals). At least two clean traces were used to calculate the averaged Mmax.

Statistical comparisons were performed using two-way repeated measures ANOVA, with Condition (pre vs. post) as the within-subject factor and Group (A, B, C) as the between-subject factor. When significant effects were detected (*p* < 0.05), Bonferroni post hoc tests were applied. H-reflex latency was analyzed using the same ANOVA structure. Data normality was verified using the Shapiro–Wilk test, and homogeneity of variance was assessed via Levene’s test. The results indicated no violation of normality (for all tests: *W* > 0.90, *p* > 0.08). The assumption of sphericity was inherently satisfied due to the two-level within-subject factor (pre vs. post), as confirmed by Mauchly’s test (*W* = 1.00, *p* = 1.00).

## 3. Results

Hmax/Mmax ratios were successfully recorded in 22 athletes before and 15 min after the treadmill run to exhaustion. The individual data showed considerable variability in both baseline values and the magnitude of post-exercise change (Figure 1). To summarize these patterns and account for baseline differences, participants were grouped into three categories (A, B, and C) based on their pre- and post-exercise Hmax/Mmax ratios.

**Figure 2:**
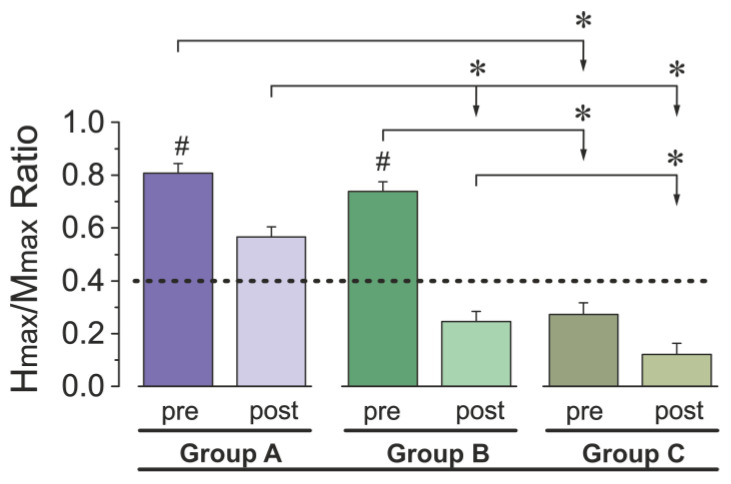
Averaged Hmax/Mmax ratios before (pre) and after (post) treadmill running across three groups (A, B and C). Bars represent mean values ± SEM. The black dashed line indicates the threshold value of 0.40 used for group classification. #—indicates significant differences between pre- and post-test values within the same group (*p* < 0.05); *—indicates significant differences between groups at the same time point (*p* < 0.05).

Across the entire sample, the Hmax/Mmax ratio decreased following the running protocol. Mean values changed from 0.61 ± 0.02 pre- to 0.31 ± 0.02 post-run (Δ = 0.30). Group-level data are presented in Figure 2: Group A showed higher baseline values (0.81 ± 0.037) and moderate reductions after exercise (0.57 ± 0.04), Group B showed substantial decreases (0.74 ± 0.04 to 0.25 ± 0.04), and Group C maintained lower values overall (0.27 ± 0.04 to 0.12 ± 0.043).

A two-way repeated-measures ANOVA confirmed these observations. There was a significant main effect of *Time* (F(1, 19) = 140.82, *p* < 0.0001, η^2^_p_ = 0.88, 95% CI [0.80, 0.90]), indicating an overall decrease in the Hmax/Mmax ratio after the exercise. A significant main effect of *Group* (F(2, 19) = 43.18, *p* < 0.0001, η^2^_p_ = 0.82, 95% CI [0.74, 0.84]) reflected stable differences between groups across time. The *Time* × *Group* interaction was also significant (F(2, 19) = 16.98, *p* < 0.0001, η^2^_p_ = 0.64, 95% CI [0.58, 0.68]), indicating that the magnitude of pre- to post-exercise change differed among the three groups.

Post hoc comparisons showed that the reduction was largest in Group B (Δ = 0.49, *p* < 0.0001), followed by Group A (Δ = 0.24, *p* = 0.003). Group C showed no statistically significant change (Δ = 0.15, *p* = 0.32). Before the exercise, Groups A and B did not differ significantly from each other (*p* > 0.05), while both had higher ratios than Group C (*p* < 0.0001). After the exercise, all three groups differed significantly (*p* < 0.05).

**Figure 3:**
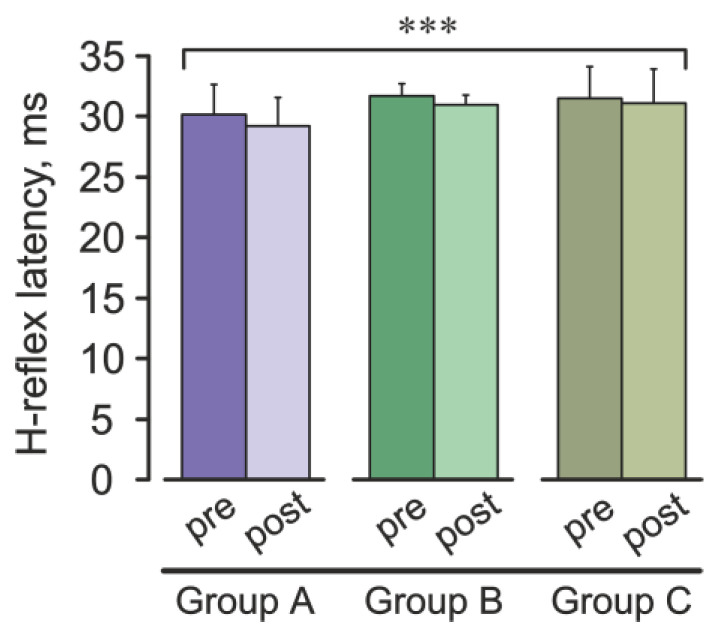
H-reflex latency values before (pre) and after (post) treadmill running across all groups (A, B and C). Bars represent mean latency ± SEM. *** *p* < 0.001, main effect of *Time*.

H-reflex latency was also examined. Mean latency decreased slightly from 31.13 ± 1.97 ms (pre) to 30.44 ± 1.97 ms (post) (Figure 3). A two-way repeated-measures ANOVA showed a significant main effect of *Time* (F(1, 19) = 15.98, *p* = 0.00077, η^2^_p_ = 0.46, 95% CI [0.41, 0.51]), but it remained within the normal physiological range. However, neither the *Group* effect (F(2, 19) = 1.54, *p* = 0.23, η^2^_p_ = 0.14, 95% CI [0.13, 0.23]) nor the *Time* × *Group* interaction (F(2, 19) = 0.75, *p* = 0.49, η^2^_p_ = 0.07, 95% CI [0.06, 0.16]) reached significance. Post hoc comparisons did not reveal significant pre- to post-exercise changes within any single group (*p* > 0.05).

Refined data on the Hmax/Mmax ratio and H-reflex latency are presented in Table 2.

Robustness checks using Holm–Bonferroni and Dunn–Šidák corrections confirmed the same outcomes as Bonferroni. For the Hmax/Mmax ratio, Group B showed the largest suppression, Group A a moderate reduction, and Group C no change. For latency, the overall reduction remained significant, with no group differences under any correction.

## 4. Discussion

This study examined spinal excitability in trained runners by assessing changes in the Hmax/Mmax ratio before and after an exhaustive running protocol. The results demonstrated substantial inter-individual variability in reflex modulation, consistent with prior work showing that H-reflex behavior depends strongly on sport type, training background, and neuromuscular demands. Endurance and resistance training have been shown to induce opposite adaptations in H-reflex gain, reflecting sport-specific modulation of spinal and supraspinal circuits [17,18,19]. Our results align with this concept by revealing three distinct patterns of reflex behavior, indicating that athletes differ markedly in how their spinal pathways respond to both acute exertion and long-term training exposure.

The post-exercise suppression observed in some participants (Group B) contrasted with the relative stability of reflex amplitude in others (Group A), suggesting that both adaptive and potentially maladaptive mechanisms may shape spinal excitability in athletic populations. Athletes with extensive endurance training often demonstrate blunted reflex modulation, possibly reflecting enhanced inhibitory control and stabilization of spinal circuits during prolonged loading [20]. Conversely, individuals with heightened spinal sensitivity or limited adaptive modulation may express more pronounced suppression following fatigue [21]. The overall decrease in amplitude is likely driven primarily by increased presynaptic inhibition of Ia afferents, which is highly responsive to metabolic and mechanical feedback from group III and IV fibers activated during exhaustive exercise [3,12]. Other inhibitory pathways, including postsynaptic and supraspinal influences [22], likely interact with presynaptic mechanisms to regulate reflex responsiveness, although isolating their individual contributions remains challenging.

Participants in Group C, characterized by low Hmax/Mmax ratios both before and after exercise, may represent a population with chronically reduced baseline spinal excitability. Two interpretations remain plausible. One is that repetitive axial loading and stretch–shortening cycles inherent in certain sports (e.g., basketball, gymnastics, hurdling) may contribute to subtle neurophysiological alterations or early mechanical stress affecting lumbar afferent pathways.

Comparable reductions in H-reflex amplitude have been reported in clinical populations such as radiculopathy and polyneuropathy [5,6], but our asymptomatic participants cannot be interpreted diagnostically. At the same time, low reflex gain may also represent an adaptive pattern: reduced spinal reflex responsiveness can enhance motor precision and limb stiffness control in high-skill tasks [23,24,25], suggesting a well-tuned inhibitory profile rather than dysfunction. Distinguishing between these scenarios requires longitudinal data and complementary diagnostic approaches such as MRI or nerve conduction studies.

Groups were defined using a threshold Hmax/Mmax ratio of 0.40, consistent with prior methodological approaches, which allowed differentiation of athletes with high, moderate, and low reflex responsiveness. Training history likely contributed to the observed variability. Group A consisted mainly of athletes with continuous running or field-sport backgrounds, whereas Group C included individuals transitioning from high-impact sports with greater spinal loading demands. Such differences in mechanical exposure and neuromuscular demands may help explain divergent baseline excitability and post-exercise responses. Understanding these sport-specific loading histories is therefore crucial for interpreting H-reflex profiles and avoiding oversimplified classification of adaptive versus potentially adverse patterns.

A modest but significant reduction in H-reflex latency was observed across the full sample, although this effect did not differ across groups and remained within physiological norms. This uniform shortening of latency likely reflects non-specific peripheral factors such as exercise-induced changes in nerve and muscle temperature [26,27]. Because the observed change remained within established normative variability, it should not be interpreted as a mechanistic marker of fatigue-related neural adaptation. Additional contributions from transient shifts in membrane excitability or neurotransmitter kinetics may also play a role [28,29,30]. The weak association between latency and amplitude changes reinforces that these parameters reflect different physiological processes: latency is more sensitive to peripheral conduction factors, while amplitude reflects central modulation and afferent–motoneuron synaptic strength.

Overall, these findings demonstrate heterogeneous modulation of spinal excitability in trained runners, with distinct group profiles and modest latency changes. While diagnostic conclusions cannot be drawn, the results highlight the relevance of H-reflex assessment for monitoring training adaptation and identifying atypical neurophysiological patterns in athletes.

Limitations. Several limitations should be acknowledged. The sample size was modest, and participants were exclusively trained runners, which may limit the generalizability of the results to other athletic populations. Moreover, the cross-sectional design precludes causal inference regarding whether reduced spinal excitability represents a risk factor or an adaptive trait. The study did not include direct imaging or neurochemical correlates to confirm structural changes in the spinal cord or peripheral nerves. Future research should incorporate longitudinal designs, combining electrophysiological monitoring with the MRI-based assessments of spinal integrity and diffusion tensor imaging to explore microstructural correlates of reflex modulation. While a statistically significant decrease in latency was observed post-exercise, this change remained within normal physiological variability and therefore its mechanistic or functional relevance should not be over-interpreted. Without concurrent nerve conduction studies or temperature measurements, the underlying mechanisms cannot be fully confirmed. Finally, the exploratory group classification based on Hmax/Mmax thresholds should be interpreted cautiously, as it requires validation in larger and more diverse cohorts.

## 5. Conclusions

This pilot study investigated spinal excitability in trained runners by analyzing Hmax/Mmax ratios and H-reflex latency before and after exhaustive running. The results revealed heterogeneous suppression of reflex amplitude, modest latency reduction, and distinct group profiles, consistent with the study objective of assessing variability in spinal modulation. Groups were defined by threshold criteria, and differences appeared related to training background, but causal interpretations cannot be drawn. While the H-reflex proved sensitive to both acute exertion and training history, its diagnostic utility remains speculative in the absence of clinical validation, and the observed latency reduction should therefore not be interpreted as a mechanistic marker of fatigue-related neural adaptation.

The pilot nature of this work limits generalizability, but the findings support further investigation into sport-specific adaptations of spinal excitability. Future studies should employ larger samples, longitudinal designs, and integration with imaging or nerve conduction measures to clarify whether reflex profiles can serve as biomarkers of adaptation or early dysfunction.

In summary, H-reflex testing offers promise as a non-invasive tool for profiling neuromuscular responses in athletes, but its role in clinical or preventive applications requires cautious interpretation and systematic validation.

## Figures and Tables

**Table 1:** Participant characteristics.

Group	Participant’s Code	Gender (Male, Female)	Athlete’s Specialization (m)	Transition History	Training Tenure in T and F
A	p01	M	1500	Soccer ⟶ Running	3 years
	p02	M	1500, 3000	—	4 years
	p03	F	800, 1500	Volleyball ⟶ Running	3 years
	p04	M	3000	Soccer ⟶ Running	5 years
	p05	M	800	—	8 years
	p06	F	200	—	7 years
	p07	F	100, 400	—	2 years
	p08	F	400	—	1 years
B	p09	M	2000 hurdles	Soccer ⟶ Running	5 years
	p10	M	800	Trekking ⟶ Running	5 years
	p11	F	2000, 3000	Soccer ⟶ Running	5 years
	p12	F	2000 hurdles	—	6 years
	p13	M	800	—	9 years
	p14	F	3000, 5000	—	9 years
	p15	M	1500	Soccer ⟶ Running	4 years
	p16	F	800, 1500	—	7 years
C	p17	F	400 hurdles	—	6 years
	p18	F	5000	Basketball ⟶ Running	4 years
	P19	M	1500, 3000	Basketball ⟶ Triathlon ⟶ Running	2 years
	p20	M	2000 hurdles	—	6 years
	p21	F	100, 200	Gymnastic ⟶ Running	6 years
	p22	F	800, 1500	—	3 years

**Table 2:** Summary chart of mean ± SEM values of Hmax/Mmax ratio and latency before and after exercise in the three participant groups.

Variable	Group A (*n* = 8)		Group B (*n* = 8)		Group C (*n* = 6)	
	Pre	Post	Pre	Post	Pre	Post
Hmax/Mmax	0.81 ± 0.04	0.57 ± 0.04	0.74 ± 0.03	0.25 ± 0.03	0.27 ± 0.04	0.12 ± 0.04
Latency (ms)	30.19 ± 2.45	29.25 ± 2.35	31.69 ± 0.99	31.00 ± 0.80	31.5 ± 2.65	31.08 ± 2.85

## Data Availability

The original contributions presented in this study are included in the article. The raw data supporting the conclusions of this article will be made available by the authors on reasonable request.

## Abbreviations

The following abbreviations are used in this manuscript:

AD/DA	Analog-to-Digital/Digital-to-Analog Converter
ANOVA	Analysis of variance
BMI	Body mass index
EMG	Electromyography
MRI	Magnetic resonance imaging
RPE	Rating of perceived exertion
SEM	Standard error of the mean
SENIAM	Surface electromyography for the non-invasive assessment of muscles
T and F	Track and field
